# Effects of intravenous administration of magnesium sulfate in propofol-based sedation for ERCP in elderly patients: a randomized, double-blind, placebo-controlled study

**DOI:** 10.1186/s12877-023-04107-6

**Published:** 2023-07-07

**Authors:** Jie Chen, Ke Qian, Chun-hong Liu, Xiao-li Li, Yan Chen, Jin-quan Wang

**Affiliations:** 1 Department of Anaesthesiology, People’s Hospital of Chongqing Banan District, Chongqing, China; 2 Department of Anaesthesiology, Southwest Hospital, Third Military Medical University, Chongqing, China; 3 Department of Anaesthesiology, The Ninth People’s Hospital of Chongqing, No. 69, Hejia Road, Jialing village, Beibei District, Chongqing, 400700 P. R. China

**Keywords:** Intravenous magnesium, Sedation, Elderly patients, Endoscopic retrograde cholangiopancreatography

## Abstract

**Background:**

Propofol-based sedations are widely used in elderly patients for endoscopic retrograde cholangiopancreatography (ERCP) procedure, but respiratory depression and cardiovascular adverse events commonly occur. Magnesium administered intravenously can alleviate pain and decrease propofol requirements during surgery. We hypothesized that intravenous magnesium was used as adjuvant to propofol might be beneficial in elderly patients undergoing ERCP procedures.

**Methods:**

Eighty patients aged from 65 to 79 years who were scheduled for ERCP were enrolled. All patients were intravenously administered 0.1 µg/kg sufentanil as premedication. The patients were randomized to receive either intravenous magnesium sulfate 40 mg/kg (group M, *n* = 40) or the same volume of normal saline (group N, *n* = 40) over 15 min before the start of sedation. Intraoperative sedation was provided by propofol. Total propofol requirement during ERCP was the primary outcome.

**Results:**

The total propofol consumption were reduced by 21.4% in the group M compared with the group N (151.2 ± 53.3 mg vs. 192.3 ± 72.1 mg, *P* = 0.001). The incidences of respiratory depression episodes and involuntary movement were less in the group M than those in the group N (0/40 vs. 6/40, *P* = 0.011; 4/40 vs. 11/40, *P* = 0.045; respectively). In the group M, the patients experienced less pain than those in the group N at 30 min after the procedure (1 [0–1] vs. 2 [1–2], *P* < 0.001). Correspondingly, the patients’ satisfaction was clearly higher in the group M (*P* = 0.005). There was a tendency towards lower intraoperative heart rate and mean arterial pressure in group M.

**Conclusions:**

A single bolus of 40 mg/kg of intravenous magnesium can significantly reduce propofol consumption during ERCP, with higher sedation success and lower adverse events.

**Trial Registration:**

ID UMIN000044737. Registered 02/07/2021.

## Background

Endoscopic retrograde cholangiopancreatography (ERCP) is a common procedure employed to diagnose and treat disorders of pancreaticobiliary pathologies [1]. With the aging of the population and increasing incidence of biliary tract diseases, elderly patients become the main population applying for ERCP [2]. Considering the technical complexities and long procedural time of ERCP, adequate sedation and pain control are required throughout the procedure for immobility, analgesia, and patient comfort [3, 4].

Propofol is the most commonly used hypnotic agent in sedation during endoscopic procedures for its advantages of fast onset and quick recovery. However, propofol may cause respiratory depression and cardiovascular events, which are easier to appear in cases of old age and high-speed administration of propofol [5, 6]. Moreover, high doses of propofol may cause dose-dependent hemodynamic instability in older or feeble patients. Therefore, it is essential to minimize the dosage of propofol to prevent cardiopulmonary complications associated with sedation, especially in the elderly, by combining with an adjuvant medication. Although benzodiazepines or opioids combined with propofol can reduce the consumption of propofol, elderly patients usually feature a higher overall body fat content than younger patients which may delay the metabolism of lipid-soluble propofol, opioid, and benzodiazepines [7]. Therefore, repeated doses of such agents in elders may result in a prolonged sedation state or respiratory depression. Dexmedetomidine is another effective adjuvant that can be co-administered with propofol for ERCP, but may results in a longer recovery time and greater hemodynamic instability in the elderly [8]. Thus, it is significative to validate a non-opioid adjuvant for elderly patients with propofol sedation to reduce propofol consumption and related side effects.

Magnesium is a non-specific calcium channel inhibitor and a non-competitive N-methyl-D-aspartate (NMDA) receptor antagonist, which has analgesic and mild sedative properties [9]. It has been demonstrated that intravenous administration of magnesium sulfate can reduce the overall use of intraoperative propofol and the postoperative analgesic requirement effectively in various types of surgery [10, 11]. These findings implied that magnesium sulfate may be used as a promising adjuvant drug for ERCP sedation due to its analgesic and sedative properties. However, until now, there is no report regarding the effects of magnesium sulfate as an adjuvant in elderly patients undergoing propofol sedation for ERCP procedures.

We hypothesized that the antinociceptive, anesthetic and central sedative effects of magnesium might be beneficial in elderly patients undergoing ERCP procedures. This prospective study was designed to evaluate the effects of intravenous magnesium administered as adjuvant to propofol during ERCP on propofol consumption and peri-procedural adverse events.

## Methods

### Ethics

This was a prospective, randomized and double-blinded study performed in the endoscopic unit of People’s Hospital of Chongqing Banan District between August 13, 2021 and March 25, 2022. The protocol had been registered (UMIN000044737) in UMIN Clinical Trials Registry (UMIN-CTR). Ethical approval for the study (No. 011-2021) was obtained from the Ethics Committee of People’s Hospital of Chongqing Banan District, Chongqing, China (Chairperson: Prof. Yang Tang) and written informed consent was obtained from each participant.

### Inclusion and exclusion

Adult patients of American Society of Anesthesiologists (ASA) physical status I through III aged between 65 and 79 years of either sex scheduled for ERCP under sedation were included in this study. Exclusion criteria were severe cardiac, lung, renal, neurological, or liver diseases, hypotension (systolic blood pressure < 90mmHg) or uncontrolled hypertension (systolic blood pressure > 170mmHg, diastolic blood pressure > 100mmHg), pre-existing hypoxemia (SpO2 < 90%), known hypersensitivity to any of the drugs that would be used in the study, and a history of adverse events with prior sedation. Additionally, patients who had taken any sedative drug within the previous 24 h and those who refused participation were excluded.

### Randomization and blinding

Eligible patients were randomly assigned to either a magnesium sulfate group or a normal (0.9%) saline group at a ratio of 1:1 using a computer-generated sequence. Group assignments were placed into opaque, sealed, consecutively numbered envelopes by staff who was not involved in the trial. The envelopes were opened just prior to ERCP by a certified registered anesthesia nurse who also prepared the drugs in coded syringes according to the order number indicating the group of assignment, and she/he was not associated with the management of patients and data collection and analyses. All patients, endoscopist, anesthetist, and data collector were blinded to the group assignment.

### Sedation protocol

The patients were fasted routinely for overnight, and received 5ml of oral 2% lidocaine hydrochloride mucilage and topical spray oropharyngeal anesthesia with 4% lidocaine as premedication 20 min prior to the start of the sedation. Then the patients were placed in prone position with their head on the right side and administered intravenous isotonic saline solution at a rate of 6–8 ml/kg/h throughout the procedure via a 20-gauge intravenous catheter that was inserted in peripheral veins of the left upper limb and connected via extension tubes to an infusion pump (BeneFusion SP5 Ex, Mindray Bio-Medical Electronics Ltd., Nanshan, Shenzhen, China) for propofol infusions. The patients were monitored according to our hospital standards including continuous monitoring of heart rate (HR), peripheral blood oxygen saturation (SpO2), end-tidal carbon dioxide (EtCO2), and mean arterial pressure (MAP) measured every 5 min. Supplemental oxygen was administered via a nasal cannula at 4 L/min throughout the procedure.

Prior to the beginning of the sedation, all patients were intravenously administered 0.1 µg/kg sufentanil and then divided into two groups: group M and group N. In group M, 40 mg/kg magnesium sulfate diluted with normal saline to a total volume of 100 ml was administered for 15 min. The rationale for the dosage of the magnesium sulfate regimen was based on previous studies [11, 12]. Patients in group N received an equal volume of normal saline as a placebo. All patients received standard sedation with propofol. An initial bolus dose of 1 mg/kg propofol was administered over 30 s followed by a continuous intravenous infusion of propofol at a maintenance dose of 2 mg/kg/h. The ERCP procedure was performed after disappearance of the eyelash reflexes by two experienced endoscopists who had performed at least 500 procedures respectively. The Ramsay Sedation Scale (RSS, 1: patients feeling anxious and agitated or restless, or both; 2: patients feeling co-operative, oriented, and tranquil; 3: patients responding to commands only; 4: patients exhibiting brisk response to light glabellar tap or loud auditory stimulus; 5: patients exhibiting sluggish response to light glabellar tap or a loud auditory stimulus; 6: no response to stimulus) was used to assess the level of sedation throughout the procedure, and a score of 5 or higher was targeted for the procedure [13]. In case of a score lower than 5 or patient expressed discomfort (involuntary movement, grimaces) and difficulty in maneuvering the endoscope, 0.25 mg/kg of propofol was used in the form of a bolus as rescue drugs. Meanwhile, the propofol infusion rate was upregulated by 0.5 mg/kg/h, and repeated the process if necessary. If the patient suffered from respiratory depression (SpO2 < 90% for > 10 s), the essential respiratory supports such as chin/jaw lifting or assisted mask ventilation were immediately provided until SpO2 reverted to normal. In case of hypotension (MAP < 70 mmHg or the decline reached 20% of the basal value) which persisted for more than 1 min, rehydration and intravenous injection of 40 µg phenylephrine was administered. If bradycardia (HR < 50 beats/min) occurred, intravenous injection of 0.5 mg atropine was performed.

At the end of the procedure, all infusion drugs were stopped immediately, and the dose of the agents were recorded. After the procedure, patients were transferred to the post-anesthesia care unit (PACU) for monitoring. The modified Aldrete Score was used to assess overall recovery of patients. Patients were allowed to be transferred to wards when a score of 9 or more was identified.

### Study endpoint

The primary outcome was total propofol consumption. The main secondary outcomes were: procedure time (defined as the time from the insertion of the endoscope to the extraction of the endoscope); sedation time (defined as the time from administration of propofol to the end of the procedure); the incidence of adverse events (e.g., respiratory depression, hypotension, bradycardia, involuntary body movement, postoperative nausea and vomiting (PONV), lethargy, and arrhythmia); the essential vital signs (such as MAP, HR, SpO2) and RSS score at the following time points: before induction (T0), at the beginning of the procedure (T1), 10 and 20 min after beginning of the procedure (T2 and T3), after endoscope removal (T4). Additional secondary endpoints were: awakening time (defined as the time from the end of the procedure to spontaneous eye opening); recovery time (defined as the time from the end of the procedure to achieving a modified Aldrete score ≥ 9); the pain score at 30 min after procedure (measured by a visual analog scale (VAS) from 0 to 10; the higher the score, the more severe the pain); the satisfaction of endoscopists and patients (assessed by a VAS from 0 to 10; the higher the score, the better the satisfaction). Two anesthesiology residents assessed and recorded the clinical data.

### Data analysis

The sample size was calculated based on the primary outcome of this study, the total cumulative dose of propofol during ERCP procedure. A placebo-controlled pilot study of 20 patients showed the mean ± standard deviation (SD) of administered dose of propofol during procedure was 207.4 ± 73.7 mg. We anticipated a 20% difference in propofol needs between the placebo group and the magnesium sulfate group (less propofol consumption in magnesium sulfate group). By setting a two-sided alpha of 0.05 and a beta error of 0.1, a minimum of 34 patients in each group would be required to allow the detection of a difference between groups with a power of 90%. In our study, 40 elderly patients were recruited in each group for possible dropouts.

Statistical analysis was performed by SPSS version 25.0 (SPSS Inc, Chicago, Illinois, USA). Continuous variables, including physiological parameters, total cumulative dose of propofol, duration of procedure, awakening and recovery time, pain score at 30 min after ERCP, as well as satisfaction of the endoscopists and patients were presented as mean ± SD or medians with the upper and lower quartiles, whereas categorical variables were expressed as frequency and percentages. Shapiro-Wilk’s test was used to assess the data distribution. Continuous variables were compared between groups by the independent samples *t*-test (normal distribution) or Mann-Whitney U test (non-normal distribution), and categorical variables were compared between groups by the Chi-square test or Fisher exact test. Analysis of variance (ANOVA) was used to analyze repeated-measured variables such as MAP, HR, SpO2 and RSS. A *P*-value < 0.05 was set as statistically significant for all analyses.

## Results

**Fig. 1:**
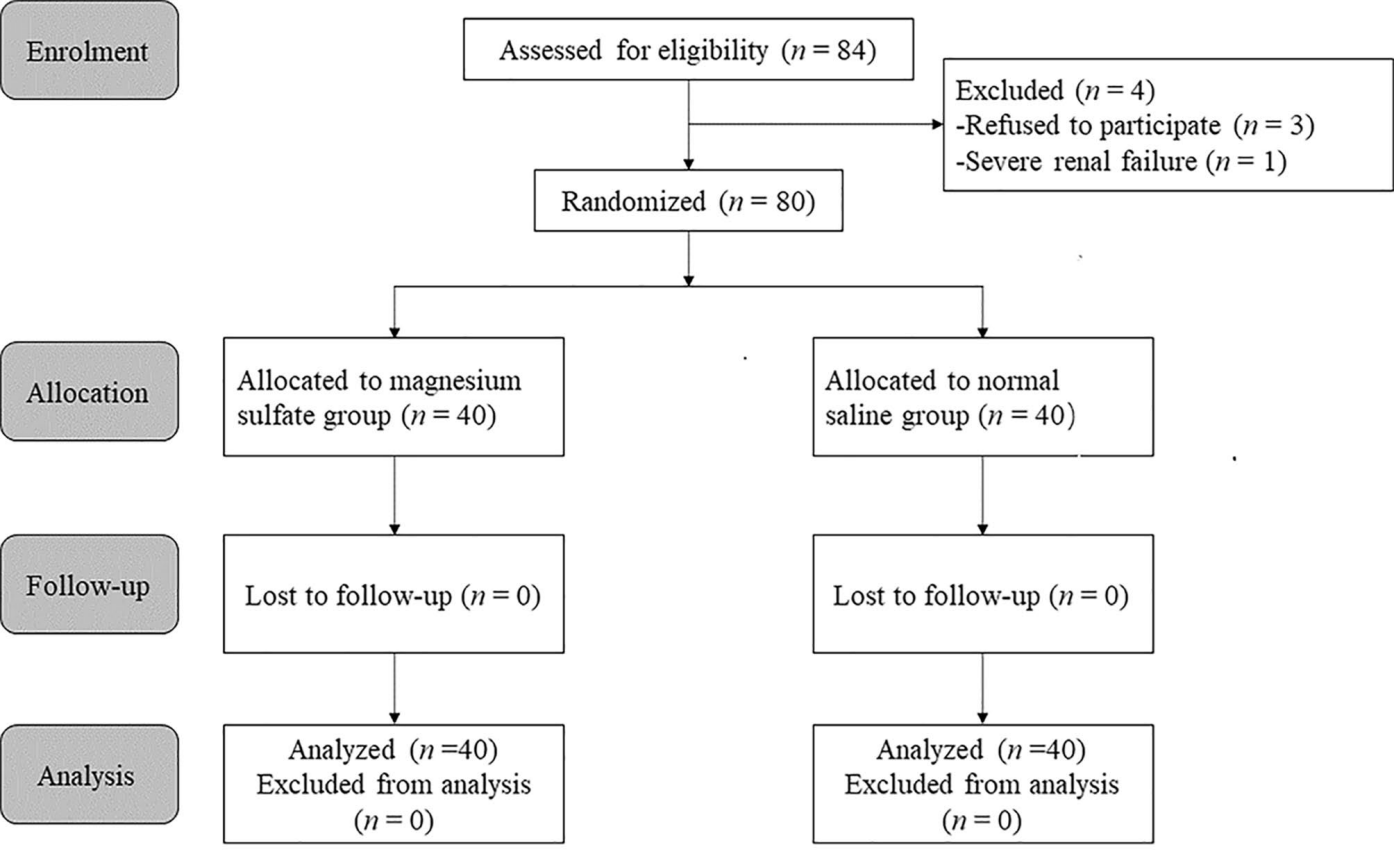
Flow diagram of design and recruitment of participants

A total of eighty-four patients were enrolled initially in the present study. Of these, four patients were excluded due to severe renal failure (*n* = 1) and refusal to sign informed consent (*n* = 3). Ultimately, a total of 80 elderly patients (35 males) were evaluated (Fig. 1). There were no significant differences in the demographic characteristics and ERCP procedure details between the two groups (*P* > 0.05 for all; Table 1).

**Table 1:** Demographic characteristics and procedure details

Variable	Group N (n = 40)	Group M (n = 40)	*P* value
Age, years	70.1 ± 6.0	70.4 ± 5.5	0.426
Sex (male/female), *n*	17/23	18/22	0.822
BMI, kg·m^− 2^	22.3 ± 3.6	23.3 ± 3.1	0.525
ASA status I/II/III, *n*	12/18/10	10/21/9	0.792
Comorbidities, *n* (%)			
Hypertension	19 (47.5)	18 (45.0)	0.832
Diabetes	9 (22.5)	12 (30.0)	0.446
Cerebrovascular disease	2 (5.0)	1 (2.5)	0.556
Chronic pulmonary disease	5 (12.5)	7 (17.5)	0.531
Combined other	2 (5.0)	1 (2.5)	0.556
Snoring, *n* (%)	16 (40.0)	13 (32.5)	0.485
ERCP indications, *n* (%)			
Bile duct stone	31 (77.5)	30 (75.0)	0.793
Benign obstruction	4 (10.0)	2 (5.0)	0.396
Malignant obstruction	5 (12.5)	8 (20.0)	0.363
Interventional ERCP, *n* (%)	38 (95.0)	39 (97.5)	0.556
Duration of sedation, min	52.0 ± 21.9	47.8 ± 19.8	0.381
Duration of ERCP, min	47.0 ± 21.9	42.5 ± 19.4	0.348

Data are expressed as mean ± SD, or number of patients (percent). Group N, normal saline group; Group M, magnesium sulfate group; BMI: body mass index; ASA, American Society of Anesthesiologists; ERCP, endoscopic retrograde cholangiopancreatography

Intravenous magnesium significantly decreased the propofol consumption by 21.4% during the ERCP procedure (*P* = 0.001; Table 2). The total amount of propofol requirements for the magnesium sulfate and saline group subjects were 151.2 ± 53.3 mg and 192.3 ± 72.1 mg, respectively. Additionally, although the awakening time was significantly delayed in the group M when compared with the group N (*P* = 0.020; Table 3), the recovery time were similar in the two groups (*P* = 0.056; Table 3).

**Table 2:** Perioperative adverse events

Variable	Group N (n = 40)	Group M (n = 40)	*P* value
Hypotension episodes	11 (27.5)	6 (15.0)	0.172
Bradycardia episodes	2 (5.0)	4 (10.0)	0.396
Respiratory depression episodes	6 (15.0)	0 (0.0)	0.011
Involuntary movement	11 (27.5)	4 (10.0)	0.045
PONV			
Nausea	1 (2.5)	2 (5.0)	0.556
Vomiting	0 (0.0)	0 (0.0)	1.000
Lethargy	1 (2.5)	1 (2.5)	1.000
Arrhythmia	0 (0.0)	0 (0.0)	1.000

Data are expressed as number of patients (percent). Group N, normal saline group; Group M, magnesium sulfate group; PONV, postoperative nausea and vomiting

**Table 3:** Data related to sedation and recovery

Variable	Group N (n = 40)	Group M (n = 40)	*P* value
Total dose of propofol, mg	192.3 ± 72.1	151.2 ± 53.3	0.001
Awakening time, min	4.9 ± 2.9	6.3 ± 3.2	0.020
Recovery time, min	9.0 ± 3.5	10.7 ± 4.2	0.056
Pain score 30 min after ERCP	2 (1–2)	1 (0–1)	< 0.001
Endoscopists’ satisfaction	9 (8–10)	10 (9–10)	0.054
Patients’ satisfaction	10 (9–10)	10 (10–10)	0.005

Data are expressed as mean ± SD, or medians with the upper and lower quartiles. Group N, normal saline group; Group M, magnesium sulfate group; ERCP, endoscopic retrograde cholangiopancreatography

None of the patients required analgesic rescue during postoperative evaluation. But the patients in the group M experienced less pain than those in the group N at 30 min after the procedure (1 [0–1] vs. 2 [1–2], respectively; *P* < 0.001; Table 3). Meanwhile, the patients’ satisfaction was clearly higher in the group M when compared with the group N (*P* = 0.005; Table 3), but there were no statistically differences between the two groups regarding endoscopists’ satisfaction (*P* = 0.054; Table 3).

**Fig. 2:**
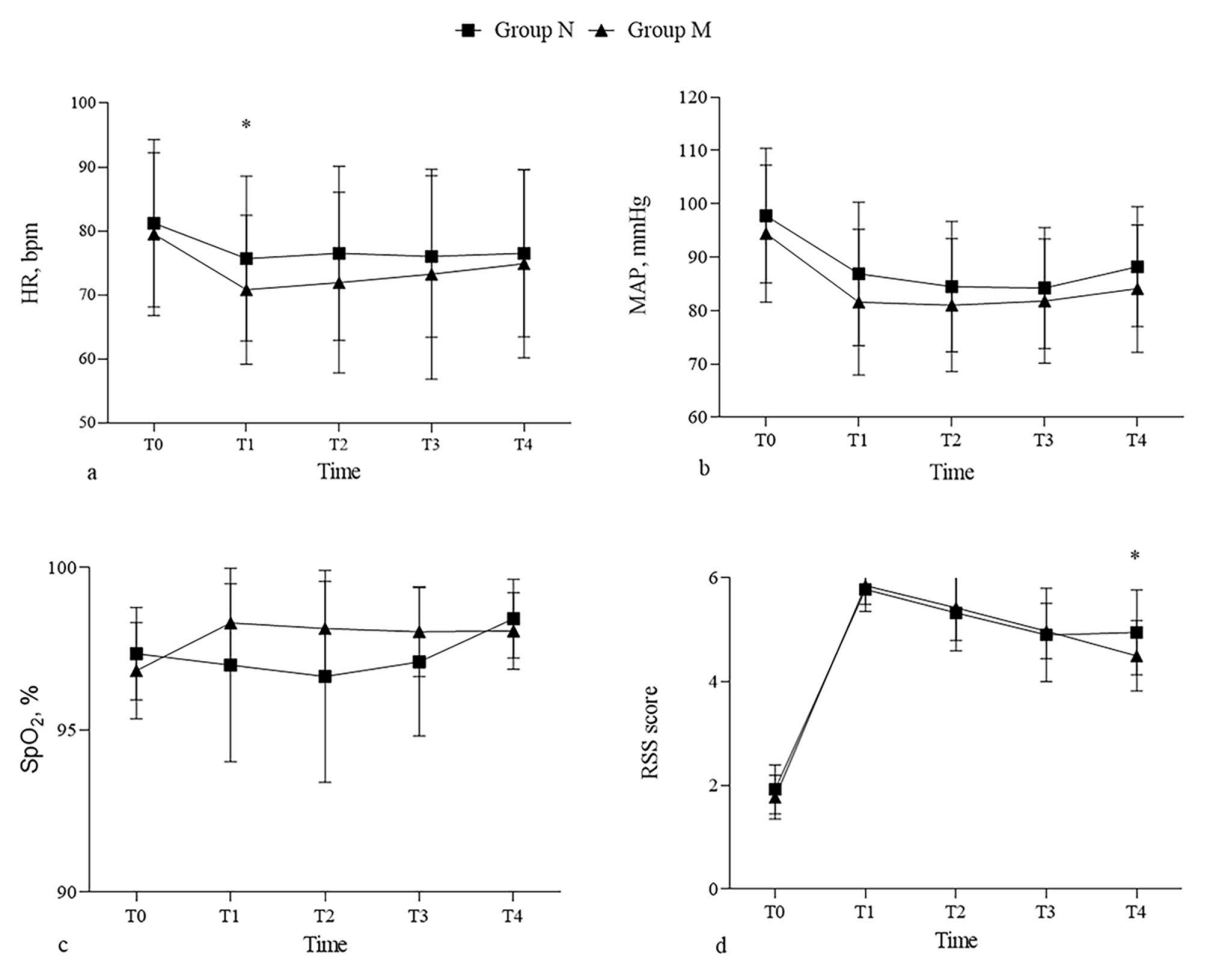
Hemodynamic parameters and RSS scores of the patients at different time points. **a**, HR. **b**, MAP. **c**, SpO2. **d**, RSS score. MAP, mean arterial pressure; HR, heart rate; SpO2, saturation of peripheral oxygen; RSS, Ramsay Sedation Scale. * Significant at *P* < 0.05

The MAP and peripheral blood oxygen saturation were not significantly different at any time point between the two group (Fig. 2b and c). The group M demonstrated lower HR values than the group N in almost all measurements, especially at time point T1, a significant decrease in the HR values with the magnesium administered was observed (*P* = 0.034; Fig. 2a). Also, the RSS score of the group M was significantly decreased compared with that of the group N at time point T4 (*P* = 0.018; Fig. 2d).

Table 2 shows the incidence of perioperative adverse events. Hypotension occurred in 11 patients (27.5%) in the group N and 6 patients (15%) in the group M (*P* = 0.172). Two patients in the group N and four patients in the group M demonstrated bradycardia, which were successfully treated with atropine alone (*P* = 0.396). Six patients (15%) developed desaturation in the group N, while no hypoxia occurred in the group M (*P* = 0.011). No endotracheal intubation or supraglottic airway device was necessary in any patient. Involuntary body movement was statistically less in the group M compared with the group N (4/40 vs. 11/40, respectively; *P* = 0.045). There were no significant differences in PONV, lethargy, and arrhythmias between the two groups (*P* > 0.05 for all; Table 2).

## Discussion

ERCP is a gold standard diagnostic and treatment method for patients with biliary and pancreatic diseases which are common in the elderly. Elders are often comorbid with cardiopulmonary diseases. Studies have shown that the high prevalence of cardiopulmonary complications may be associated with mortality, brain injury, myocardial ischemia, and the risk of mechanical ventilation during perioperative period [14–16]. Adequate sedation for ERCP procedure directly affects the operation time and success [3, 4]. Although propofol is postulated to be the most appropriate anesthetic agent for deep sedation for ERCP because of its strong sedation, rapid onset of action and lack of accumulation after drug withdrawal, it can lead to suppression of protective reflexes and various complications including respiratory depression and hemodynamic fluctuations in a dose-dependent manner, especially in elderly patients [2, 5, 6]. Therefore, it is necessary to find a suitable adjuvant to reduce the propofol consumption and minimize the risk of propofol sedation-related complications.

Intravenous magnesium is used to treat preeclampsia, eclampsia, and arrhythmia in clinical practice, but it has also been demonstrated to reduce the requirements for inhalation anesthetics, opioids and propofol during general anesthesia in various types of surgery [10–12, 17]. In addition, several previous studies have shown that intravenous magnesium had an opioid- and/or propofol-sparing effect and could attenuate the postprocedure pain intensity and the analgesic requirements in gastric neoplasm or colonoscopy [18, 19]. These results are consistent with our findings. In this study, we found that by given magnesium as a single bolus of 40 mg/kg before ERCP procedure reduces intraoperative propofol requirements by 21.4% without compromising the satisfaction score of endoscopists. It is meant that the reduction of sedative dose did not affect the endoscopists’ working conditions. Meanwhile, our data demonstrated that intravenous magnesium also led to a significant improvement in postoperative pain scores and patient satisfaction. The effects of magnesium on propofol consumption and perioperative analgesic requirements could be related to the anxiolytic and sedative properties of magnesium, but the exact mechanism for such effects remain unknown. Theoretically, magnesium inhibits the calcium channel activation and antagonizes NMDA receptors in the central nervous system and consequently modified the anesthetic effects [9, 11, 17, 19]. Another mechanism involve that magnesium inhibits the release of catecholamines through reduced sympathetic outflow, which may decrease peripheral nociceptor sensitization or the stress response to surgery [17, 19]. However, these mechanisms do not explain the decrease in propofol dosage, which is independent of the reduction in requirements for analgesic. Thus, the interaction between magnesium and propofol remains further study.

In current study, magnesium was only applied before the procedure. The predefined target level of sedation was maintained with propofol infusion alone. It was observed that although the duration of consciousness recovery was delayed by maximum 84 s with magnesium administration, the duration of PACU stay was not prolonged compared with that in saline group. As a consequence, the lower doses of magnesium combined with low dose propofol may not cause the clinically significant oversedation effect. Moreover, our results demonstrated that intravenous administration of magnesium could relieve respiratory depression in older patients during ERCP procedure. In addition, though there was no significant difference in peripheral blood oxygen saturation between magnesium and saline groups, we still found a tendency that oxygen saturation in magnesium group was slightly higher. One possible explanation might be that magnesium significantly decreased the requirement for propofol during the procedure. Propofol is associated with a high risk of respiratory depression in a dose-dependent manner in elders [20]. Therefore, respiratory depression caused by propofol was also significantly attenuated.

It is well known that magnesium might induce hypotension and bradycardia by directly inhibiting cardiac and vascular sympathetic activities and indirectly depressing catecholamine release [11, 21]. Our results showed that there was a tendency of lower intraoperative HR and MAP in magnesium group, even though it failed to reach statistical significance. In group analysis, the HR and MAP both decreased during the procedure but did not exceed 20% compared to the baseline values. Meanwhile, we did not observe a significant difference in either hypotension or bradycardia episodes between magnesium and saline groups. The reason for this observation might be that the sample size was insufficient to reveal the difference between the two groups because this study was designed for the assessment of the total propofol consumption during procedure as a primary outcome. On the other hand, magnesium was reported to be associated with lesser hemodynamic and ST segmental changes and it could play a role as a cardioprotective agent in coronary artery disease patients [19, 22]. Thus, moderate magnesium administration may have more benefits than risks in elderly patients with possible coronary artery disease.

There is a possibility of hypermagnesemia following magnesium administration. Studies have shown that magnesium-related adverse effects such as lethargy, nausea or vomiting, hypotension, bradycardia, and respiratory depression may occur when serum magnesium concentration exceeds 2 mmol/L [23, 24], which indicated that the safe upper limit of magnesium concentration in serum level was below 2 mmol/L. In the study conducted by Gao et al. [25], in patients undergoing hysteroscopy who received magnesium of 50 mg/kg and then 15 mg/kg/h continuous infusion until the end of the procedure, postoperative serum magnesium concentration was below the safe upper limit, maximally 0.96 mmol/L. Muir and colleagues [26] reported that infusion of 4 g of magnesium sulfate resulted in a plasma magnesium concentration below 1.8 mmol/L. In contrast, in the present study, the requirement of magnesium infusion was 2.4 ± 0.4 g during the procedure. Therefore, a single total dose of 40 mg/kg magnesium intravenously in this study may be safely.

This study has several limitations. Firstly, we did not measure serum magnesium concentrations before and after magnesium administration. However, it was previously reported that there is very little correlation between intra- and extracellular magnesium concentration [11, 17]. Thus, the serum magnesium concentration does not accurately indicate the magnesium levels of the tissues. Doses of magnesium similar to ours also were not shown any serious complications attributed to magnesium administration [19]. Secondly, we used RSS scores and the operator’s subjective evaluation to titrate the depth of sedation for ERCP, instead of the bispectral index or Narcotrend for two main reasons. One reason is that our hospital does not have a bispectral index or narcotrend monitor, because Chinese law does not require that patients under general anesthesia be equipped with a deph of anesthesia monitor. Another reason is that these monitoring methods have been reported to be potentially inaccurate during surgery [27, 28]. Jang and colleagues [29] have found that a lower dose of propofol is required in the presence of bispectral index monitoring. Therefore, the corresponding results are limited without using these monitoring methods during the procedure. Lastly, this was a single center study. Although the present study evaluated the effect of magnesium sulfate as an adjuvant with propofol for sedation in elderly patients during ERCP, the results of the study cannot be generalized for other age brackets and surgery types. Further research with a larger sample size and a multicenter model is warranted.

## Conclusions

A single bolus of 40 mg/kg of intravenous magnesium can significantly reduce propofol requirements and decrease the incidence of respiratory depression and involuntary body movement. Furthermore, it alleviates the postprocedure pain and allows high patient satisfaction and lower adverse events. Therefore, magnesium could be used as an effective and safe adjuvant agent for ERCP sedation in elderly patients. More research is needed to evaluate and modify the ERCP sedation strategy for elderly patients by using intravenous magnesium.

## Data Availability

The de-identified data for individual participants underlying our results can be accessed with approval from the corresponding author 6 months after publication. The study protocol, statistical analyses, and clinical study report will also be available.

## Abbreviations

ERCP: endoscopic retrograde cholangiopancreatography
NMDA: non-competitive N-methyl-D-aspartate
ASA: American Society of Anesthesiologists
HR: heart rate peripheral
SpO2: blood oxygen saturation
EtCO2: end-tidal carbon dioxide
MAP: mean arterial pressure
RSS: ramsay sedation scale
PACU: post-anesthesia care unit
PONV: postoperative nausea and vomiting
VAS: visual analog scale
SD: standard deviation
ANOVA: Analysis of variance
